# Phosphodiesterase 4 Inhibitor Roflumilast Ameliorates Polytrauma-Induced Acute Kidney Injury by Attenuating HMGB1 and Renal Inflammatory Pathways in Rats

**DOI:** 10.3390/ijms27177696

**Published:** 2026-08-28

**Authors:** Caroline Gusson Shimoura, Dustin M. Kneifel, Heaven D. Sessions, Jeanette Rocha, Kassandra Gonzalez, Emily M. Corbin, Venkata Yellepeddi, Brian J. Kirkwood, Jose Salinas, Andrew D. Meyer, Zhangsheng Yang

**Affiliations:** 1 Expeditionary Medical Systems Department, United States Army Institute of Surgical Research, 3698 Chambers Pass, Fort Sam Houston, TX 78234, USA; 2 Research Support Directorate, United States Army Institute of Surgical Research, Fort Sam Houston, TX 78234, USA; 3 Division of Clinical Pharmacology, Department of Pediatrics, Spencer Fox Eccles School of Medicine, University of Utah, Salt Lake City, UT 84132, USA; 4 Long School of Medicine, The University of Texas Health Science Center, San Antonio, TX 78229, USA

**Keywords:** phosphodiesterase inhibitor, roflumilast, trauma, hemorrhage, acute kidney injury, inflammation

## Abstract

Traumatic injury remains a critical global health challenge, with polytrauma frequently leading to multi-organ failure and mortality. A severe complication of polytrauma is acute kidney injury (AKI), which is driven by a massive systemic inflammatory response following the initial insult. This inflammation is exacerbated by the upregulation of phosphodiesterase 4 (PDE4) enzymes; therefore, PDE4 inhibition represents a rational therapeutic strategy. Roflumilast is an FDA-approved PDE4 inhibitor currently used for chronic obstructive pulmonary disease (COPD). This study evaluated the hypothesis that roflumilast can mitigate inflammation and prevent AKI in a polytrauma rat model. Anesthetized male Sprague–Dawley rats were randomly assigned to either a vehicle control group (*n* = 9) or a roflumilast treatment group (0.2 mg/kg, *n* = 10). All animals underwent a polytrauma protocol—including soft tissue injury, fibula fracture, and pressure-controlled hemorrhage—followed by whole blood resuscitation and a 72-h observation period. Rats treated with roflumilast demonstrated higher survival rates compared to injury controls (70% vs. 33%). Furthermore, roflumilast treatment significantly lowered blood urea nitrogen (BUN, *p* < 0.05) and renal levels of Kidney Injury Molecule-1 (KIM-1, *p* < 0.05). Roflumilast also significantly reduced levels of circulating High Mobility Group Box 1 (HMGB1) (*p* < 0.05) and the proinflammatory cytokine macrophage inflammatory protein-1 alpha (MIP-1α) (*p* < 0.05). These findings suggest that roflumilast is a promising therapeutic agent for mitigating trauma-induced AKI.

## 1. Introduction

Traumatic injury represents a major global health burden affecting military personnel, veterans, and civilian populations. Worldwide, trauma accounts for approximately five million deaths annually and imposes substantial logistical and financial demands on military medical systems, the U.S. Department of Veterans Affairs, and civilian healthcare systems [1]. Severe trauma can precipitate subsequent organ failure, frequently progressing to multiple-organ failure (MOF) [2,3]. MOF is characterized by simultaneous injury to two or more organ systems and commonly results from blast exposure, hemorrhagic shock, soft-tissue and extremity trauma, burns, penetrating injuries (e.g., gunshot wounds), motor vehicle collisions, or falls [4,5,6]. These multifactorial insults generate profound systemic physiological stress and initiate complex inflammatory and hemodynamic cascades.

Acute kidney injury (AKI) is a frequent and clinically significant complication of trauma and a primary driver of MOF, prolonging hospitalization and increasing morbidity and mortality [7,8]. The development of AKI following trauma is multifactorial, primarily driven by renal hypoperfusion, ischemia–reperfusion injury, and systemic inflammation [9,10,11,12]. Increasing evidence suggests that inflammation plays a central role in the pathogenesis of trauma-induced AKI, as polytrauma triggers a robust systemic inflammatory response [13]. The kidney is particularly vulnerable to inflammatory and microvascular dysregulation due to its high metabolic demand and dense microvascular network [14]. The infiltration of activated immune cells into renal tissue further amplifies local cytokine production, disrupts microvascular integrity, and exacerbates hypoxic injury [9,15,16]. Currently, there are no U.S. Food and Drug Administration (FDA)-approved pharmacological therapies specifically aimed at treating or mitigating AKI by targeting inflammation in polytrauma.

During the systemic inflammatory response in trauma, the increase in the activity of phosphodiesterases (PDEs) amplifies inflammatory signaling pathways and promotes immune cell activation, leading to tissue injury [17]. Elevated PDE reduces intracellular cyclic adenosine monophosphate (cAMP) levels, which is a key regulatory pathway involved in modulating inflammation. Intracellular cAMP acts as a critical anti-inflammatory second messenger that limits immune cell activation, cytokine production, and leukocyte adhesion. PDEs, particularly PDE4, rapidly hydrolyze cAMP and thereby constrain its anti-inflammatory effects [18]. In systemic inflammatory states such as trauma and sepsis, increased PDE activity can further deplete intracellular cAMP levels, amplifying inflammatory signaling pathways and promoting immune cell activation [19]. Therefore, targeting this pathway represents a mechanistically attractive strategy to attenuate inflammation-driven organ damage.

It is well-established that pharmacological inhibition of PDE4 prevents the degradation of cAMP, which suppresses pro-inflammatory cytokine release, reduces leukocyte activation, and stabilizes endothelial function, collectively limiting tissue injury and microvascular dysfunction [20,21]. Roflumilast is a selective PDE4 inhibitor approved by the FDA for the treatment of chronic obstructive pulmonary disease (COPD) [22], and it has demonstrated systemic anti-inflammatory effects beyond its pulmonary benefits [23,24]. For example, in experimental models of systemic inflammation and sepsis, roflumilast reduced renal injury, decreased immune cell infiltration, and inhibited apoptosis [25,26]. These findings suggest that repurposing roflumilast represents a promising strategy to mitigate inflammation-driven kidney injury following trauma. In the present study, we evaluated the therapeutic effects of roflumilast in a clinically relevant rat model of polytrauma characterized by systemic inflammation and renal injury. We hypothesized that selective inhibition of PDE4 by roflumilast would attenuate trauma-induced inflammation, reduce renal injury, and improve overall outcomes.

## 2. Results

### 2.1. Roflumilast Increases Survival in a Rat Polytrauma Model

**Figure 1:**
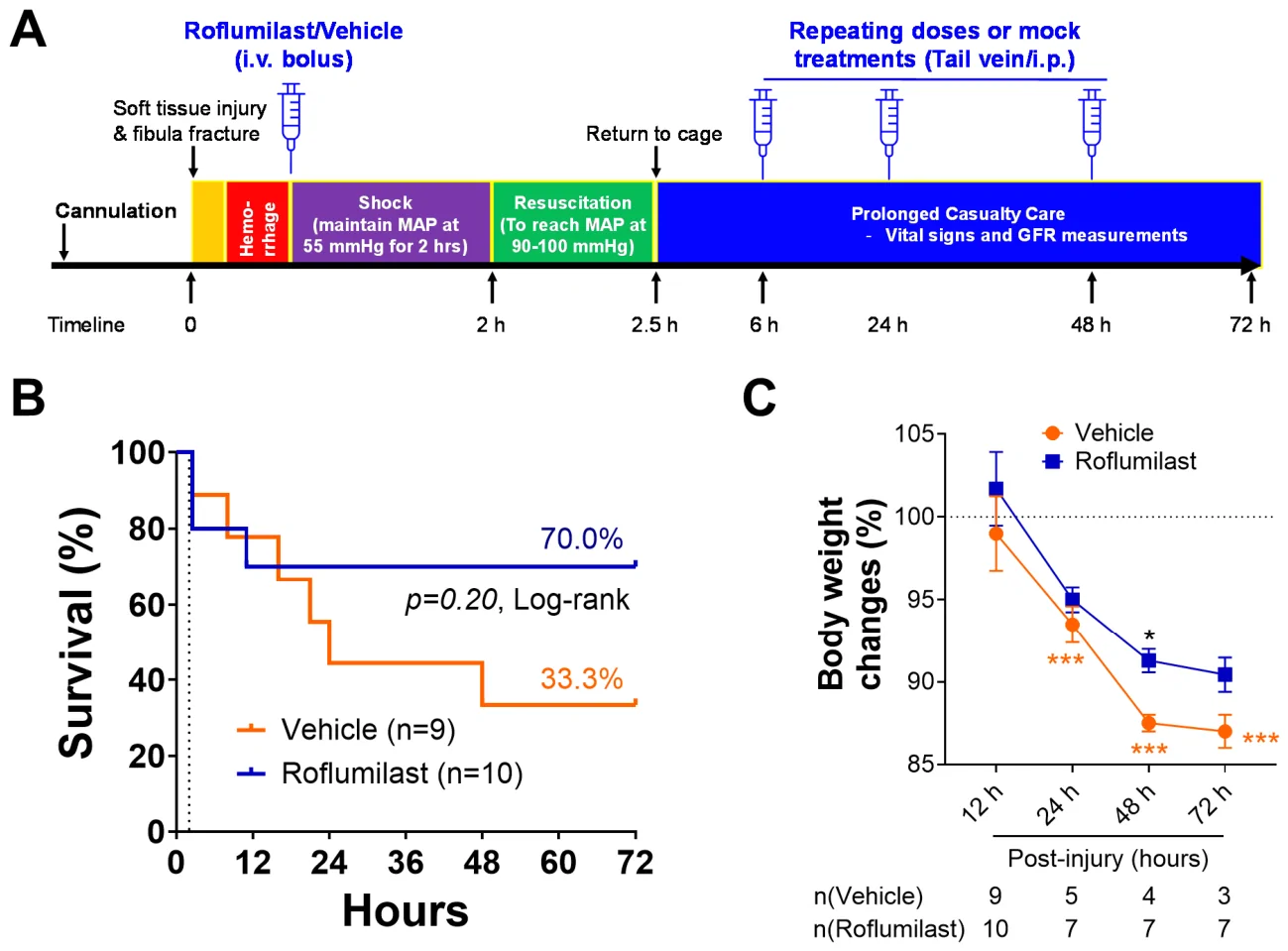
Effect of roflumilast on survival in a rat model of polytrauma. (**A**) Experimental timeline and schematic design for polytrauma rats treated with roflumilast or vehicle control. All rats underwent anesthetized polytrauma injury consisting of a soft tissue injury, fibula fracture, and pressure-controlled hemorrhage. Then, they received an intravenous (i.v.) injection of either roflumilast (0.2 mg/kg) or vehicle (saline at an equivalent volume), followed by a period of hemorrhagic shock maintained for a total of 2 h from initial injury and donor blood resuscitation for 30 min. The animals were allowed to recover and return to their cages. Three subsequent doses of roflumilast treatment (each 0.2 mg/kg) or vehicle control were administered at 6, 24, and 48 h after injury. All animals were monitored for 72 h post-injury. (**B**) Treatment with roflumilast showed a trend toward increased survival compared to the injury control group, suggesting that PDE4 inhibition may confer a protective effect following polytrauma and hemorrhagic injury. Log-rank test. *p* = 0.2. (**C**) Polytrauma animals that received roflumilast treatment had significantly attenuated weight loss compared to animals that received the vehicle treatment, especially at 48 h post-injury (*, *p* < 0.05, ***, *p* < 0.001).

To evaluate the therapeutic effects of PDE4 inhibition, roflumilast was administered intravenously following hemorrhage and at 6, 24, and 48 h after injury (Figure 1A). Survival analysis demonstrated an increased survival rate in the treated group compared to injured controls (70% vs. 33.3%, *p* = 0.2; Figure 1B). In the untreated injury control group, mortality was highest within the first 24 h, with one additional death occurring at 48 h post-injury. In contrast, in the roflumilast treatment group, two animals died before 12 h following injury, after which the survival rate stabilized. Furthermore, animals treated with roflumilast exhibited significantly better body weight maintenance compared to untreated animals, with a significant difference observed at 48 h post-injury (87.00 ± 1.00% vs. 90.43 ± 1.04%, *p* < 0.05) (Figure 1C).

### 2.2. Roflumilast Treatment Improves Metabolic Status

**Figure 2:**
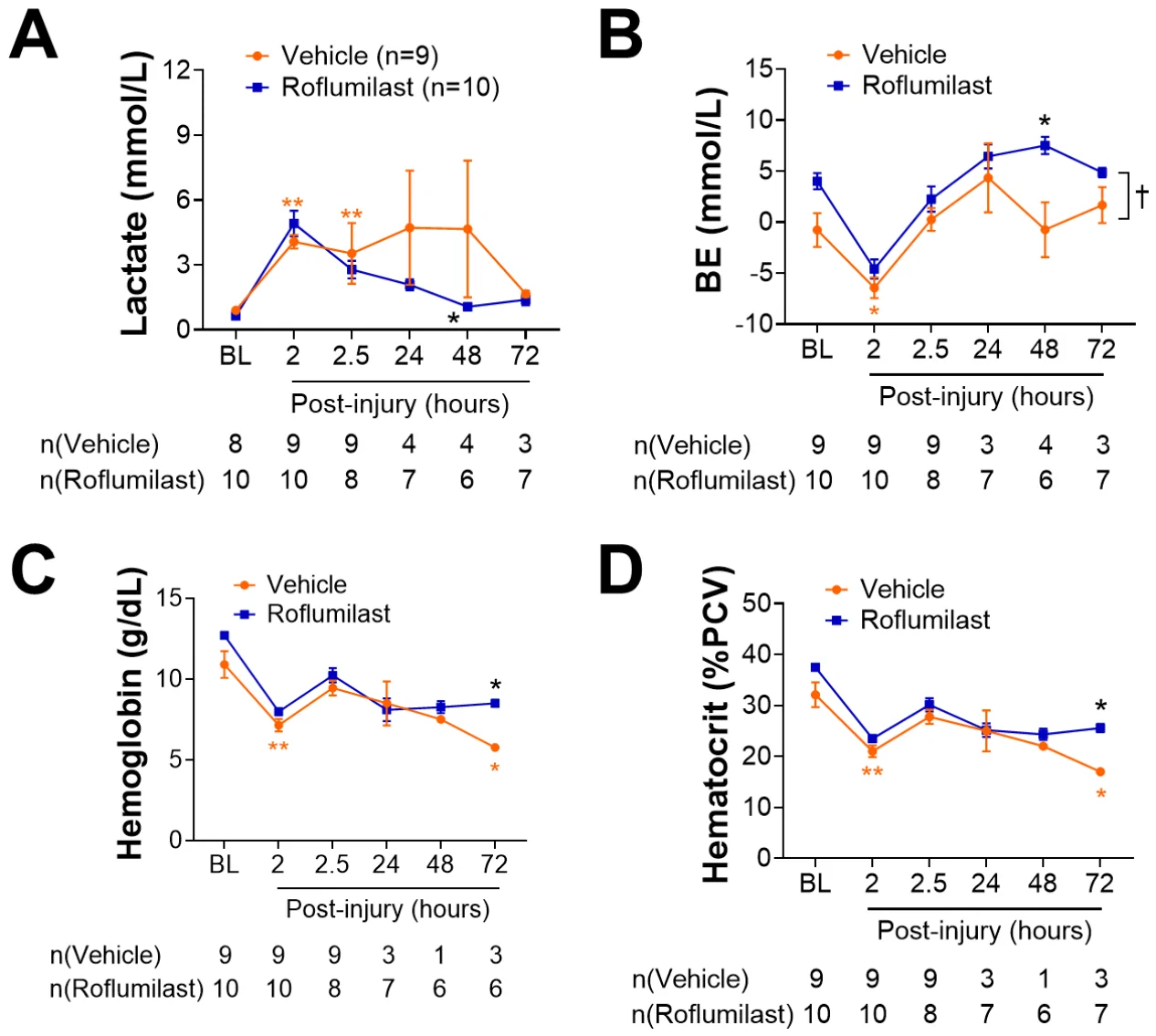
Roflumilast treatment attenuates metabolic and hematologic dysfunction following polytrauma. Plasma lactate, Base Excess (BE), hemoglobin, and hematocrit were measured at Baseline (BL), 2, 2.5, 24, 48, and 72 h after polytrauma injury. (**A**) Roflumilast treatment blunted the injury-induced rise in plasma lactate, with a significant reduction observed at 48 h post-injury. (**B**) BE levels were significantly increased in the roflumilast group, while hemoglobin (**C**) and hematocrit (**D**) levels were significantly higher in roflumilast treatment groups compared to the vehicle controls at the 72-h time point. Statistical analyses were performed using a linear mixed-effects model for repeated measures. †, *p* < 0.05 polytrauma vs. roflumilast. To compare individual time points to baseline within the injury group, a paired Wilcoxon signed-rank test was used (*, *p* < 0.05; **, *p* < 0.01).

Lactate levels serve as critical indicators of tissue hypoperfusion and injury [27]. Our rat model of polytrauma is characterized by increased blood lactate levels, suggesting severe injury and metabolic stress. Indeed, in vehicle control animals lactate levels significantly increased at all timepoints measured after injury, starting at 2 h with sustained high levels until 72 h (BL: 0.9 ± 0.4; 2 h: 4.1 ± 0.9; 2.5 h: 3.5 ± 4.2; 24 h: 4.7 ± 5.2; 48 h: 4.7 ± 6.3; 72 h: 1.7 ± 0.2 mmol/L, *p* < 0.05 all compared to BL). Treatment with roflumilast significantly decreased lactate levels at 48 h post-injury compared to vehicle control (4.7 ± 6.3 mmol/L vs. 1.1 ± 0.2 mmol/L, *p* < 0.05) (Figure 2A). Base Excess (BE), a marker of acid-base balance, decreased at 2 h post-injury in both roflumilast-treated and control animals. At the 48 h time point, BE was significantly increased in the roflumilast group compared to control (7.5 ± 0.85 vs. −0.75 ± 2.69 mmol/L), as shown in Figure 2B.

Hemoglobin and hematocrit were decreased post injury in both control and roflumilast treated animals (hemoglobin control BL: 10.9 ± 0.8; 2 h: 9.5 ± 0.5 g/dL vs. roflumilast BL: 12.7 ± 0.1; 2 h: 10.2 ± 0.3 g/dL; hematocrit control BL: 32.1 ± 2.4; 2 h: 27 ± 1.4% PCV vs. roflumilast BL: 37.5 ± 0.5; 2 h: 30.1 ± 0.9; 72 h: 25.6 ± 0.9% PCV). At 72 h post-injury, both hematocrit and hemoglobin were significantly increased in the roflumilast group compared to control at the same time point (Figure 2C,D) (hemoglobin 5.8 ± 2 vs. 8.5 ± 0.2 g/dL; hematocrit 17 ± 0.6 vs. 25.6 ± 0.9% PCV). Additionally, roflumilast treatment increased pH at the end of shock and 24 h after injury, increased glucose at the end of shock and end of resuscitation, respectively), and significantly stabilized HCO_3_ and TCO_2_ levels at 48 h post-injury (Table 1).

### 2.3. Roflumilast Effects on Renal Injury and Renal Function

**Figure 3:**
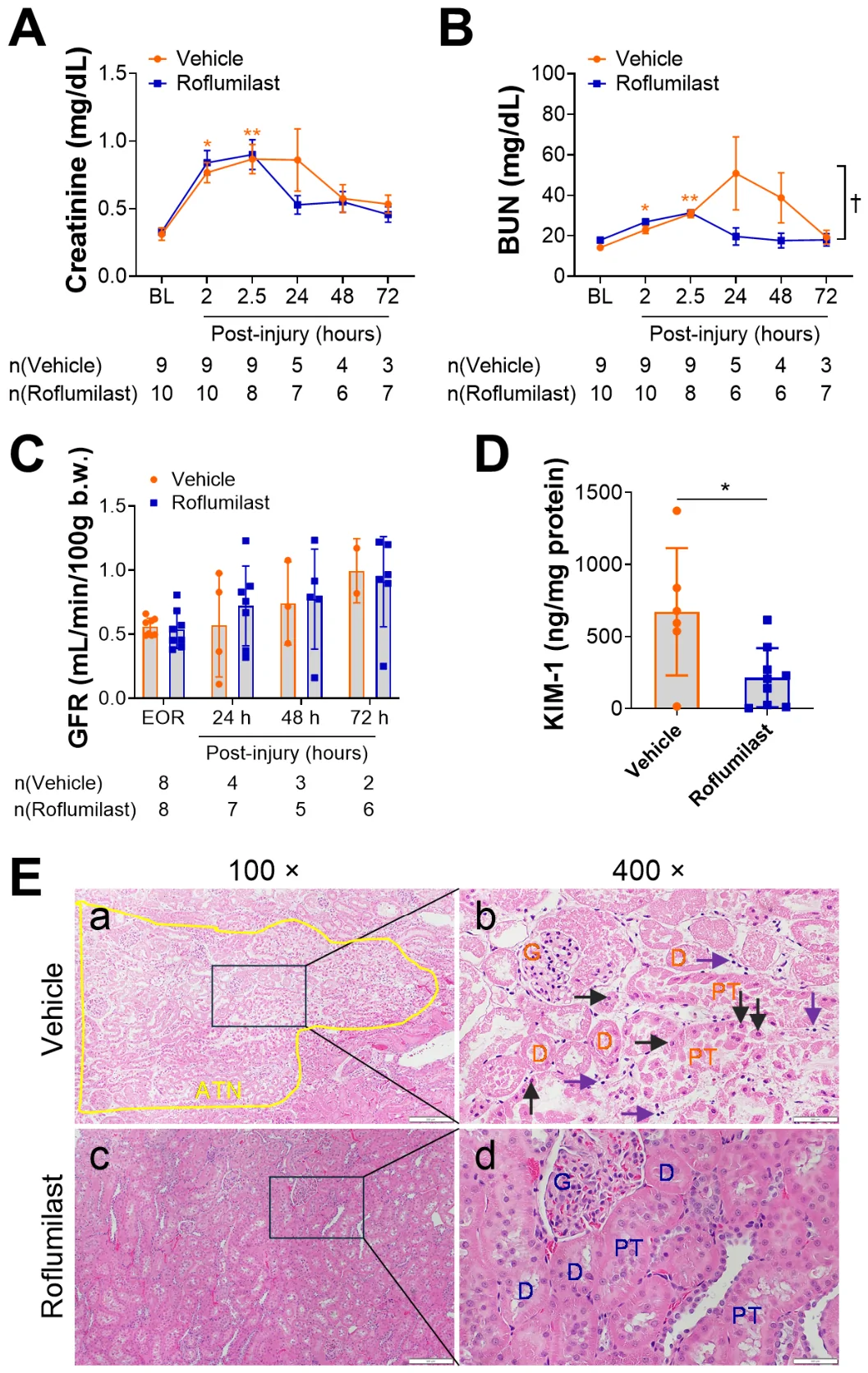
Roflumilast treatment improves renal function in a polytrauma rat model. Plasma creatinine (**A**) and blood urea nitrogen (BUN) (**B**) levels were quantified via iSTAT at baseline (BL) and at 2, 2.5, 24, 48, and 72 h following polytrauma injury. A significant reduction in BUN was observed in the roflumilast treatment group compared to vehicle controls. (**C**) Glomerular filtration rate (GFR) in vehicle- and roflumilast-treated animals at the end of resuscitation (EOR, 2.5 h post-injury) and at 24, 48, and 72 h post-injury. (**D**) Kidney Injury Molecule-1 (KIM-1) protein levels measured in renal homogenates from vehicle- and roflumilast-treated animals at 72 h post-injury. (**E**) Representative photomicrographs of H&E-stained histologic sections of the kidney at 72 h post-injury. Rats subjected to polytrauma exhibited tubular epithelial necrosis and disruption of the tubular architecture (**a**,**b**); these pathological changes were attenuated following roflumilast treatment (**c**,**d**). To compare individual time points to baseline within the injury group, a paired Wilcoxon signed-rank test was used (*, *p* < 0.05; **, *p* < 0.01) and †, *p* < 0.05 for the least-squares means between the groups.

Our polytrauma model is characterized by significant renal injury, evidenced by an increase greater than 1.4-fold in plasma creatinine level from baseline. Notably, plasma creatinine levels increased significantly as early as 2 h post-injury in vehicle control group compared to baseline (BL: 0.3 ± 0.05 vs. 2 h: 0.8 ± 0.07 mg/dL, *p* < 0.001) and remained elevated throughout the 72-h observation period (EOR: 0.9 ± 0.11; 24 h: 0.9 ± 0.23; 48 h: 0.6 ± 0.1; 72 h: 0.5 ± 0.07 mg/dL) (Figure 3A). The blood urea nitrogen (BUN) levels were also increased significantly at 2 h following injury in the control group (BL: 14.2 ± 1.4 vs. 2 h: 23 ± 1.8 mg/dL, *p* < 0.01) and remain elevated for the 48 h timepoint (EOR: 30.9 ± 1.9; 24 h: 50.8 ± 18; 48 h: 38.8 ± 12.6; 72 h: 19.3 ± 3.4 mg/dL) (Figure 3B).

Treatment with roflumilast reduced plasma creatinine levels at 24 h compared with the vehicle control group; however, this reduction did not reach statistical significance (0.9 ± 0.23 vs. 0.5 ± 0.07 mg/dL, *p* = 0.7). Furthermore, roflumilast treatment significantly decreased overall BUN levels when compared to the vehicle control (†, *p* = 0.008), indicating a partial attenuation of renal impairment.

Although roflumilast treatment did not significantly alter glomerular filtration rate (GFR) compared to vehicle controls (Figure 3C), it resulted in a significant reduction in tissue levels of Kidney Injury Molecule-1 (KIM-1, 672.5 ± 18.4 vs. 214.8 ± 205.1 ng/mg protein, Figure 3D, *p* < 0.05). Histological analyses further demonstrated that roflumilast attenuated renal tissue damage. While vehicle control animals exhibited severe acute tubular necrosis (ATN, yellow encircled region), with necrosis characterized by retention of architecture with loss of differential staining and tubular epithelial nuclear karyolysis (black arrows), fibrocyte and structural support cell nuclear pyknosis (purple arrows), and tubular epithelial degeneration (not pictured) (Figure 3(Ea,Eb)), roflumilast treatment preserved tubular viability and morphology, with intact architecture, including proximal and distal tubules, and retention of differential staining (Figure 3(Ec,Ed)).

### 2.4. Roflumilast Treatment Decreases HMGB1 and MIP-1α Following Polytrauma

**Figure 4:**
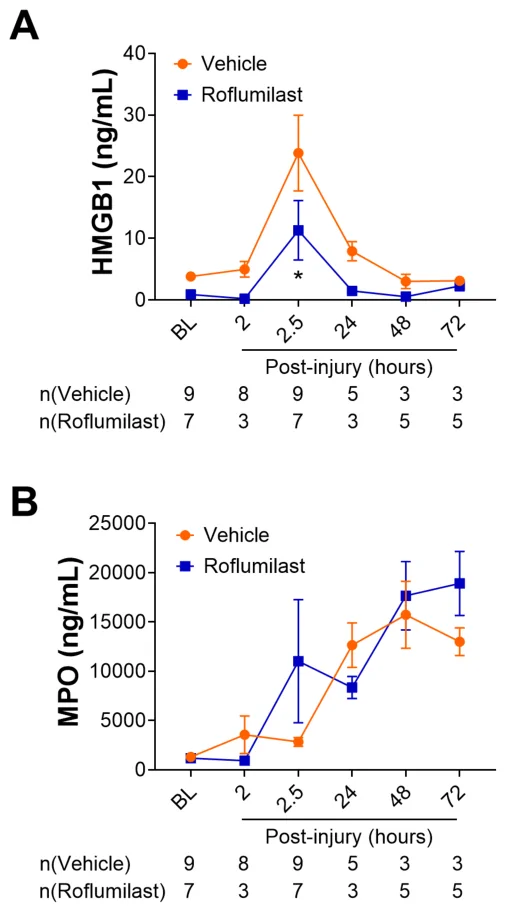
Roflumilast decreases the systemic level of HMGB1. The levels of HMGB1 (**A**) and MPO (**B**) were quantified in the plasma at Baseline (BL), 2.5, 24 h, 48 h, and 72 h after polytrauma injury. A significant reduction in HMGB1 was observed at 2.5 h post-injury in roflumilast-treated animals compared to the vehicle control. Statistical analyses were performed using a paired Wilcoxon signed-rank test (*, *p* < 0.05).

**Figure 5:**
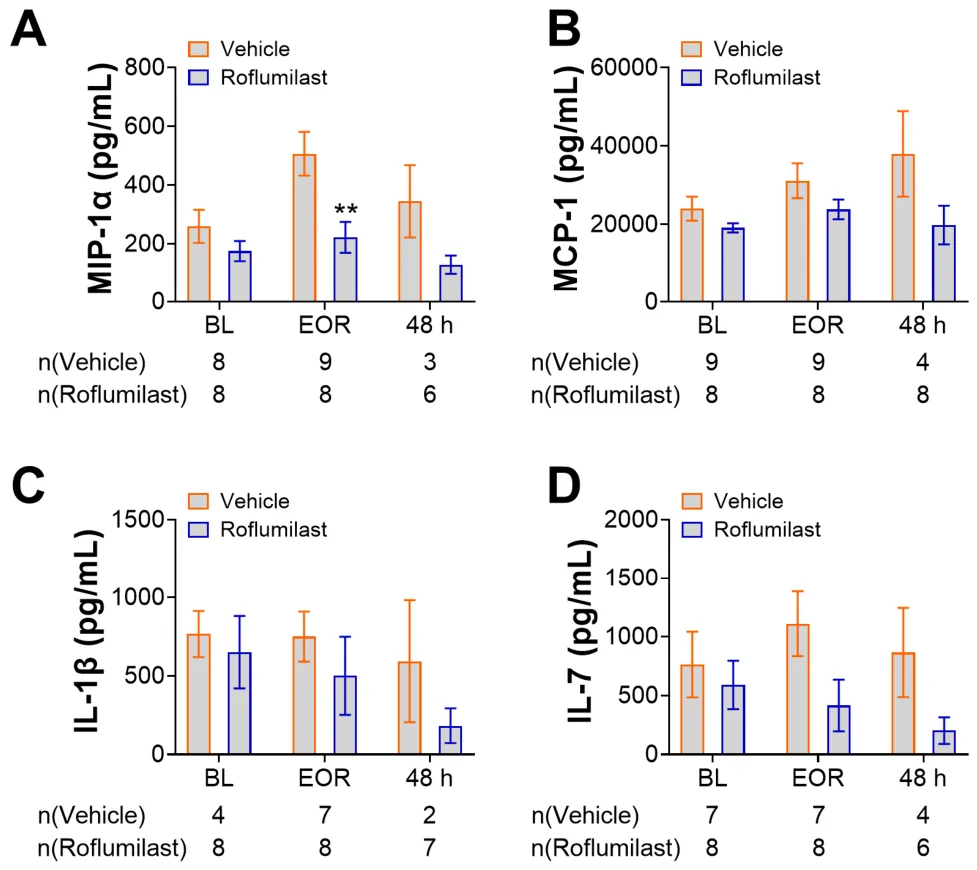
Roflumilast reduces systemic inflammatory response. The pro-inflammatory cytokines, including MIP-1α (**A**), MCP-1 (**B**), IL-1β (**C**), and IL-7 (**D**), were measured in plasma by Bio-Plex at baseline (BL), end of resuscitation (EOR, at 2.5 h post-injury), and 48 h post-injury. Statistical analyses were performed using the paired Wilcoxon signed-rank test (**, *p* < 0.01).

Roflumilast treatment significantly reduced circulating plasma HMGB1 levels at 2.5 h post injury compared to vehicle control (3.02 ± 1.2 vs. 0.51 ± 0.2 ng/mL, *p* < 0.05; Figure 4A) and MIP-1α was also significantly lower in the roflumilast treatment group at 2.5 h post injury (506.6 ± 74.4 vs. 221.8 ± 30.9 pg/mL, *p* < 0.05; Figure 5A).

## 3. Discussion

Our findings indicate that roflumilast represents a promising therapeutic strategy for the management of trauma-induced organ failure. Post-trauma administration of roflumilast improved survival rates and significantly attenuated acute kidney injury (AKI), as demonstrated by reduced tissue levels of KIM-1 and BUN. Furthermore, roflumilast treatment mitigated the elevation of lactate and significantly improved outcomes. Roflumilast also suppressed inflammatory markers, significantly reducing circulating levels of HMGB1 and MIP-1a. Collectively, these data demonstrate the therapeutic potential of roflumilast in reducing mortality and mitigating inflammation-induced multi-organ dysfunction following severe trauma.

Roflumilast is an FDA-approved drug indicated for the treatment of chronic obstructive pulmonary disease (COPD) [23]. Beyond its approved clinical indication, the drug has also demonstrated significant therapeutic potential in several preclinical disease models. For instance, in rat models of cecal ligation and puncture (CLP)-induced polymicrobial sepsis, roflumilast reduced pro-inflammatory cytokine levels, lipid peroxidation, oxidative stress, and lung injury, while improving intestinal barrier function [28,29]. Similarly, in sepsis-induced AKI models, roflumilast suppressed inflammatory mediators—including TNF-α, IL-6, and IL-1β—which inhibited cellular apoptosis and attenuated kidney injury [25,30]. In a rat model of thoracic contusion, treatment with roflumilast promoted functional recovery, reduced neurodegeneration, increased anti-inflammatory cytokine expression, and activated pathways associated with angiogenesis and vascular remodeling [31]. In spinal cord injury models, roflumilast reduced reactive oxygen species (ROS) levels and improved motor function [32]. Additionally, long-term treatment with roflumilast improved learning, reduced anxiety-like behavior, and increased pro-BDNF expression in diabetic rats [33]. It also reduced pulmonary inflammation and edema in models of acute lung injury [30] and septic shock [34]. In the current study, it is important to note that while roflumilast significantly reduced HMGB1 and MIP-1α, it did not significantly alter other evaluated markers, including MCP-1, IL-1β, IL-7, and MPO. This suggests that rather than a broad, universal suppression of systemic inflammation, roflumilast exerts a more selective immunomodulatory effect in this polytrauma model, focusing primarily on key upstream danger signals and specific chemokine pathways. To our knowledge, this is the first report of the specific protective effects of roflumilast on mortality and kidney injury in a combat-relevant polytrauma model.

Our data demonstrate that mortality patterns differ significantly between groups. Untreated animals primarily died within the first 24 h post-injury. Conversely, in the roflumilast-treated cohort, early mortality was limited to the initial 12 h, with sustained survival thereafter. This temporal shift in survival is interesting, as it may suggest that early administration of roflumilast enhances physiological resilience immediately following severe injury. While these preliminary findings are of interest for prolonged casualty care (PCC) environments where evacuation is often delayed, claiming that roflumilast extends the clinical “golden hour” remains speculative. Our study did not directly test delayed administration windows, and the overall survival difference did not reach statistical significance. Therefore, these survival patterns should be interpreted cautiously as an exploratory hypothesis that requires validation in larger, dedicated therapeutic window trials [35]. By improving early metabolic stability, roflumilast may potentially expand the therapeutic window for life-saving interventions.

In our polytrauma model, controlled hemorrhage induced substantial plasma volume loss and profound physiological stress. Hemorrhage-associated sympathetic activation promotes systemic vasoconstriction and redistribution of interstitial fluid, contributing to the alterations in hemoglobin and hematocrit despite whole-blood resuscitation as observed in our study. Notably, at 72 h post-injury, hemoglobin and hematocrit levels were significantly increased in the treated group compared to untreated polytrauma controls. This delayed increase suggests possible restoration of oxygen-carrying capacity and enhancement of tissue oxygen delivery [36,37]. We hypothesize that the improvement in oxygenation, indicated by the increase in hemoglobin and hematocrit levels, likely contributed to reduced lactate accumulation, enhanced survival, and the attenuation of renal hypoxia. Consistent with this interpretation, our study showed that roflumilast treatment lowered lactate levels, increased survival, and mitigated renal injury, as evidenced by the reductions in kidney injury markers. The protective effect of roflumilast on the kidneys under these conditions highlights its potential to counteract the deleterious renal consequences of severe hemorrhage and shock.

The beneficial effects of roflumilast are likely mediated primarily through PDE4 inhibition and downstream inflammatory pathways, resulting in potent anti-inflammatory activity [20]. In our study, PDE4 inhibition significantly decreased circulating HMGB1 and MIP-1α levels and demonstrated a downward trend in IL-1β and IL-7 concentrations while decreasing kidney injury, suggesting that decreased inflammation led to organ protection. It is well-established that trauma triggers an immediate immune response, in which damage-associated molecular patterns (DAMPs) are released and initiate an extensive downstream inflammatory cascade. Typically, DAMPs—which include HMGB1 [38], extracellular histone [39], cold-inducible RNA-binding protein [40], and others—promptly activate the host inflammatory response. If these immune responses go unchecked, they can develop into a severe cytokine storm, systemic inflammatory response syndrome (SIRS), or persistent inflammation, immunosuppression, and catabolism syndrome (PICS) [41,42,43,44]. Indeed, our group previously identified HMGB1 as a prognostic biomarker that correlates with organ failure, such as acute respiratory distress syndrome (ARDS) [45], AKI [46], and MOF [46]. The significant reduction in HMGB1 and MIP-1α levels observed here indicates that roflumilast may contribute to early proinflammatory inhibition and subsequent organ protection in our model. However, further studies are warranted to investigate whether this inhibition of inflammation is directly linked to PDE inhibition and cAMP regulation.

Additional mechanisms may also contribute to the protective effects of roflumilast. Previous studies suggest that roflumilast reduces reactive oxygen species (ROS), lipid peroxidation, and mitochondrial dysfunction, potentially through attenuation of ferroptosis and oxidative stress pathways [32]. Beneficial effects have also been demonstrated across multiple organ systems, including protection against hepatic injury, preservation of intestinal barrier integrity, reduction in pulmonary inflammation and edema, and attenuation of septic AKI [28]. Collectively, these findings support a broad organ-protective role for roflumilast, mediated predominantly through modulation of inflammation and oxidative stress. We therefore hypothesize that, in polytrauma, roflumilast preserves multi-organ function, limits progression to multiple organ failure, and ultimately improves survival.

Limitations of this study should be acknowledged. First, the relatively small number of animals limits statistical power and may reduce the ability to detect more subtle physiological and molecular effects. Importantly, our longitudinal data interpretation is subject to survivor bias driven by informative missingness. Because early mortality was significantly higher in the vehicle control group, blood and tissue samples collected at 48 and 72 h post-injury represent only the most resilient surviving animals. This drop-out of the most severely injured control casualties likely underestimates the true severity of organ injury in the untreated group at later time points, thereby conservatively narrowing the apparent therapeutic difference between the vehicle and roflumilast groups. Second, we did not directly measure tissue PDE4 activity or intracellular cAMP levels in this study. While roflumilast is a highly selective and clinically established PDE4 inhibitor [22], the lack of direct upstream cyclic nucleotide quantification means we cannot definitively rule out alternative or indirect pathways contributing to the observed nephroprotection. Third, this study was conducted exclusively in male Sprague–Dawley rats. Because estrogens are known to exert natural anti-inflammatory and organ-protective effects following hemorrhagic shock, the baseline response to injury and the subsequent therapeutic efficacy of roflumilast may differ significantly in female cohorts. Excluding female animals limits the immediate generalizability of our findings, and evaluating sex as a biological variable remains a high priority for our future research. Furthermore, this investigation was conducted in a small animal model, which, despite offering high reproducibility and mechanistic insight, does not fully recapitulate the complexity of human polytrauma and the specific conditions of prolonged casualty care. Therefore, future studies in large animal models are required to validate the therapeutic effects, pharmacokinetics, hemodynamic responses, and clinical feasibility before translation to human applications.

## 4. Materials and Methods

### 4.1. Animal Study

This research was conducted in compliance with the Animal Welfare Act, the implementing Animal Welfare regulations, and the principles of the Guide for the Care and Use of Laboratory Animals. The Institutional Animal Care and Use Committee approved all research conducted in this study. The facility where this research was conducted is fully accredited by AAALAC International.

#### 4.1.1. Study Design

The PDE4 inhibitor roflumilast was purchased from MedChemExpress (MCE, Monmouth Junction, NJ, USA) and dissolved in 1% ethanol + 9% cyclodextrin (CD) + 90% saline solution. To ensure stability, the therapeutic solution was freshly prepared immediately preceding all experiments. The male Sprague–Dawley rats (10–12 weeks old), weighing 350–550 g, were purchased from Charles River Laboratories (Wilmington, MA, USA). All individuals underwent polytrauma that included soft tissue injury, fibula fracture, and pressure-controlled hemorrhage, followed by donor blood resuscitation and a 72-h observation period. Animals were randomly assigned to two groups: vehicle control (*n* = 9) or roflumilast treatment (*n* = 10, 0.2 mg/kg). To minimize animal use, a single vehicle control group was run concurrently under identical experimental conditions to serve as a shared baseline for multiple treatment arms. Because these arms were evaluated across distinct studies, a portion of this control data has been previously published [47]. Animals in the treatment group received a therapeutic intravenous (i.v.) bolus regimen of roflumilast (0.2 mg/kg) following hemorrhage, and three additional doses at 6, 24, and 48 h post-injury by tail vein or intraperitoneal (i.p.) injection. Animals in the vehicle control group received saline in the equivalent volume to the roflumilast treatment and otherwise received the same experimental manipulations and evaluations. Vital signs, hemodynamics, blood chemistry, and tissue damage were recorded and analyzed at prescribed time points following injury. Animals were excluded from the final analysis if they died or met humane euthanasia criteria before completing the polytrauma protocol due to procedural complications (such as cannulation failure) or other unexpected deaths.

#### 4.1.2. Surgical Procedures

All the animals were given proper analgesics during the study. Buprenorphine SR-Lab (subcutaneous, 1.2 mg/kg) was given prior to the surgical cannulations, and additional buprenorphine HCl (subcutaneous, 0.01–0.05 mg/kg) was administered as needed at later time points. The animals were anesthetized and maintained at a surgical plane by inhaled isoflurane. They were placed in a supine position, and the temperature was monitored. Under sterile conditions, animals received a PE50 polyethylene catheter (Braintree Scientific, Inc., Braintree, MA, USA) into the carotid artery, and the left femoral artery and vein were catheterized using a PE20 and PE30 (Braintree Scientific, Inc., Braintree, MA, USA), respectively. The catheter in the carotid artery was used for blood pressure monitoring, the femoral artery catheter was used for hemorrhage and blood sampling, and the femoral vein catheter was used for drug infusion and fluid resuscitation. At the end of resuscitation, the catheters were closed, the animals were allowed to recover from anesthesia under continuous monitoring until they regained consciousness, and they were returned to their cages.

#### 4.1.3. Polytrauma Model

The polytrauma rat model was developed at USAISR as described previously [31,32]. Briefly, soft tissue injury was induced via clamping the retro-femoral tissue group for 30 s using an angled Kelly clamp at the first notch, and a fibula fracture was performed using a 15-gauge needle penetrated through the skin and fascia between the tibia and fibula.

After the extremity trauma, animals underwent a pump-controlled hemorrhage by withdrawing blood from the femoral artery at a rate of 0.5 mL/min (Harvard Apparatus, model PHD 22/2000, Holliston, Massachusetts, USA) until the mean arterial pressure (MAP) reached the target pressure of 55 mmHg. Blood pressure was maintained at 55 mmHg for 2 h by either withdrawing additional blood or re-infusing shed blood to maintain hemorrhagic shock.

Resuscitation was performed using whole blood (treated with 1:7 citrate–phosphate–dextrose). The infusion rate was maintained at 0.5 mL/min until MAP reached 90–100 mmHg for another 30 min. After resuscitation, the animals were returned to their cages for observation for up to 72 h post-injury. Animals were closely evaluated using pain assessment criteria 2 to 3 times daily, and they were humanely euthanized if they met the criteria for the end-of-study endpoints.

#### 4.1.4. Vital Signs and Sample Collection

Vital signs and hemodynamics were recorded by the PhysioSuite system (Kent Scientific, Torrington, CT, USA) and the PowerLab system (ADInstruments, Colorado Springs, CO, USA). Arterial partial pressure of oxygen (pO2), partial pressure of carbon dioxide (pCO2), and arterial blood gas analysis were performed using an iSTAT 300-G blood analyzer (Abbott Point of Care Inc., Princeton, NJ, USA; VetScan CG4+ and CG8+ cartridges, Abaxis Inc., Union City, CA, USA). Plasma samples were collected at baseline (BL), 2, 2.5, 24, 48, and 72 h after injury. After drawing, the plasma samples were processed, aliquoted, and stored at −80 °C. Tissue samples were collected at necropsy and either frozen and stored at −80 °C or fixed in 10% neutral buffered formalin for histologic evaluation.

#### 4.1.5. Glomerular Filtration Rate Measurement

Glomerular filtration rate (GFR) was assessed in awake, freely moving rats using a transcutaneous fluorescence detection system (MediBeacon MX GFR monitor, St. Louis, MO, USA), as described previously [33,34]. Under isoflurane anesthesia, a double-sided adhesive interface was secured to the skin overlying the kidney, and the optical sensor containing dual light-emitting diodes was mounted onto the patch. One diode emitted excitation light at 480 nm to stimulate FITC–sinistrin fluorescence, while the second detected emitted light at 521 nm through the skin.

After device placement, animals were fitted with a protective mesh wrap to maintain probe positioning. FITC–sinistrin [30–50 mg/kg/body weight (b.w.), CAT# NC1570801, Fisher Scientific, Waltham, MA, USA] was administered as an intravenous bolus via a pre-implanted vascular catheter. Then, animals were allowed to recover from anesthesia before returning to cages. Fluorescence decay was continuously recorded for 2 h. Data were downloaded for analysis of FITC–sinistrin elimination kinetics. GFR was derived from the elimination half-life (t½) of FITC–sinistrin using the relationship: GFR (mL/min/100 g body weight) = 31.26 (mL/100 g b.w.)/t½, where t½ represents the plasma half-life of the tracer.

### 4.2. ELISA Assay and Bio-Plex Assay

HMGB1 and myeloperoxidase (MPO) were quantified in the plasma using ELISA kits obtained from Tecan (CAT#30164033, Tecan, Switzerland) and Hycult Biotech (CAT#HK105, Hycult Biotech, The Netherlands), respectively, according to the manufacturer’s instructions. Monocyte chemoattractant protein-1 (MCP-1), macrophage inflammatory protein-1 alpha (MIP-1α, also known as CCL-3), IL-1β, and IL-7 were measured in the plasma using a customized Bio-Plex kit (CAT#17011106, Bio-Rad, Hercules, CA, USA) or Bio-Plex Pro™ Rat Cytokine 23-Plex (CAT#12005641, Bio-Rad, Hercules, CA, USA), and performed following the manufacturer’s recommendations. KIM-1 was quantified in tissue homogenates from frozen kidney to verify kidney injury using a commercially available ELISA kit (Cat#RKM100, R&D Systems, Minneapolis, MN, USA) according to the manufacturer’s instructions. The final values were normalized to the total protein concentration of each sample.

### 4.3. Histopathological Evaluation

The kidney tissues were collected at necropsy and fixed in 10% neutral buffered formalin for a minimum of 48 h. After fixation, transverse sections were routinely processed and embedded in paraffin. The formalin-fixed, paraffin-embedded tissue sections were cut via microtome by a certified histotechnologist at 5 µm thickness, mounted onto glass slides, and routinely stained with hematoxylin and eosin (H&E) for histopathologic evaluation. Photomicrographs of entire tissue sections from each rat were captured with a 10× or 40× objective using an Olympus microscope (Olympus, BX53, Tokyo, Japan).

### 4.4. Statistical Methods

Biochemical parameters, physiological variables, and arterial blood gases in Table 1 were presented as mean ± standard error (SD), and a Mann–Whitney U test was applied for the statistical analyses. Longitudinal data were presented as mean and standard error of the mean (SEM). A linear mixed-effects model was used for longitudinal profiles between groups; however, it should be noted that this model assumes missing data are missing at random (MAR). Because missingness in this study was driven by mortality (informative missingness), data at later time points are subject to survivor bias. To compare individual post-injury time points back to baseline within the same treatment group, the paired non-parametric Wilcoxon signed-rank test was utilized. All statistical tests were two-sided, and statistical significance was defined as *p* < 0.05. Analyses were conducted using GraphPad Prism version 10.4.0 (GraphPad Software, San Diego, CA, USA)

## 5. Conclusions

This study provides evidence that pharmacological inhibition of PDE4 using the FDA-approved roflumilast tends to improve survival and mitigate AKI in a severe polytrauma rat model. The protective effects of roflumilast are associated with the attenuation of the trauma-induced inflammatory response, as evidenced by significant reductions in key proinflammatory mediators, including HMGB1 and MIP-1α. Furthermore, the preservation of renal structure and the reduction in specific kidney damage markers, such as BUN and KIM-1, highlight the nephroprotective capabilities of roflumilast. Given that roflumilast is already clinically available, repurposing it for acute trauma care represents a highly translatable and promising therapeutic strategy to combat post-traumatic MOF.

## Figures and Tables

**Table 1:** Analysis of metabolic profiles, arterial blood gases, and hemodynamic values in vehicle control and rolfumilast-treated animals.

Parameters	Groups	BL	EOS	EOR	24 h	48 h	72 h
**MAP**	Vehicle	92.3 ± 7.9, *n* = 9	55.6 ± 0.9, *n* = 9 **	96.1 ± 4.4, *n* = 8	75.3 ± 14.2, *n* = 4 *	78.3 ± 15.6, *n* = 3	74 ± 14.7, *n* = 3
	Roflumilast	100.57 ± 8.4, *n* = 10	54.42 ± 1.7, *n* = 10 **	94.8 ± 6.1, *n* = 8	**100.6 ± 17.4, *n* = 7 ††**	90.5 ± 6.6, *n* = 7	**94.2 ± 13.7, *n* = 6 †**
**HR**	Vehicle	386.3 ± 35.5, *n* = 9	393 ± 48.9, *n* = 9	398.4 ± 25, *n* = 8	397.2 ± 35.3, *n* = 4	394.3 ± 11.7, *n* = 3	407.7 ± 29.4, *n* = 3
	Roflumilast	376.9 ± 28.2, *n* = 10	377.9 ± 49.5, *n* = 10	388.5 ± 35.3, *n* = 8	**411.5 ± 40.1, *n* = 7 ††**	378 ± 25.8, *n* = 7	368.8 ± 24.3, *n* = 6
**SPO2**	Vehicle	93.9 ± 6.1, *n* = 9	87.5 ± 7.7, *n* = 9	90.1 ± 5.5, *n* = 8	95.5 ± 5.7, *n* = 4	95.2 ± 5.6, *n* = 3	94.3 ± 6.4, *n* = 3
	Roflumilast	98.1 ± 1.9, *n* = 10	90.6 ± 9.1, *n* = 9	95.3 ± 4.2, *n* = 8	97.9 ± 1.6, *n* = 7	96.9 ± 2.7, *n* = 7	97 ± 3.5, *n* = 6
**pH**	Vehicle	7.4 ± 0.1, *n* = 9	7.3 ± 0.1, *n* = 9	7.4 ± 0.1, *n* = 9	7.1 ± 0.2, *n* = 4 **	7.5 ± 0.03, *n* = 4	7.4 ± 0.02, *n* = 3
	Roflumilast	**7.3 ± 0.05, *n* = 10** †	7.4 ± 0.13, *n* = 10 ** †	7.4 ± 0.05, *n* = 8	7.4 ± 0.04, *n* = 7 * ††	7.5 ± 0.05, *n* = 6 **	7.5 ± 0.04, *n* = 7 *
**HCO3**	Vehicle	24.7 ± 4.7, *n* = 9	20.1 ± 1.8, *n* = 9 *	25.7 ± 2.6, *n* = 8	28.3 ± 5.5, *n* = 3	23 ± 5.7, *n* = 4	26.1 ± 2.8, *n* = 3
	Roflumilast	**29.5 ± 2.6, *n* = 10** †	20.8 ± 2.3, *n* = 10 **	27 ± 3.2, *n* = 8	30.5 ± 3, *n* = 7	**30.5 ± 1.6, *n* = 6** ††	28.4 ± 1.5, *n* = 7
**TCO2**	Vehicle	25 ± 5.1, *n* = 9	21.6 ± 1.7, *n* = 9	26.9 ± 2.8, *n* = 8	29.3 ± 5.9, *n* = 3	23.8 ± 6.2, *n* = 4	27.3 ± 3.5, *n* = 3
	Roflumilast	**31.2 ± 2.7, *n* = 10** ††	21.9 ± 2.6, *n* = 10 **	28.1 ± 3.5, *n* = 8	30.4 ± 5.8, *n* = 7	**31.7 ± 1.6, *n* = 6** ††	29.6 ± 1.4, *n* = 7
**SO2**	Vehicle	90.7 ± 3.7, *n* = 9	93.7 ± 1.7, *n* = 9	94.4 ± 2, *n* = 8	96 ± 2.6, *n* = 3	96.8 ± 1.5, *n* = 4 *	92.3 ± 4, *n* = 3
	Roflumilast	**95.3 ± 2.7, *n* = 10** ††	**97.5 ± 1.9, *n* = 10** †	96 ± 3.6, *n* = 8	95.7 ± 5.2, *n* = 7	98.8 ± 0.8, *n* = 6	**98.3 ± 0.8, *n* = 7** †
**Sodium**	Vehicle	141.1 ± 5.5, *n* = 9	142.3 ± 4.1, *n* = 9	140 ± 3.9, *n* = 9	138.6 ± 2.3, *n* = 5	140.5 ± 3.1, *n* = 4	139.3 ± 2.5, *n* = 3
	Roflumilast	140.3 ± 6.7, *n* = 10	139.1 ± 6, *n* = 10	137.9 ± 3.4, *n* = 8	140.7 ± 1.6, *n* = 7	138.7 ± 1.4, *n* = 6	140.1 ± 0.7, *n* = 7
**Potassium**	Vehicle	3.7 ± 0.8, *n* = 9	4.6 ± 1, *n* = 9	5.1 ± 1.6, *n* = 9	5.02 ± 2.4, *n* = 5	4.9 ± 1.8, *n* = 4	3.9 ± 0.7, *n* = 3
	Roflumilast	4.4 ± 0.6, *n* = 10	4.4 ± 0.9, *n* = 10	9.3 ± 12.8, *n* = 8	4.4 ± 0.5, *n* = 7	4.3 ± 0.5, *n* = 6	4.2 ± 0.2, *n* = 7
**Chloride**	Vehicle	106.3 ± 5.6, *n* = 9	105.4 ± 4, *n* = 9	105.4 ± 3.2, *n* = 9	104 ± 4.1, *n* = 5	107.8 ± 1.9, *n* = 4	104.3 ± 2.1, *n* = 3
	Roflumilast	105.2 ± 5.7, *n* = 10	102.5 ± 3.2, *n* = 10	102.4 ± 2.4, *n* = 8	103.4 ± 2.4, *n* = 7	**102.3 ± 2.7, *n* = 6** †	103.3 ± 1.4, *n* = 7
**Calcium**	Vehicle	1.2 ± 0.2, *n* = 9	0.9 ± 0.4, *n* = 9 *	1.1 ± 0.2, *n* = 9	1.3 ± 0.1, *n* = 5	1.35 ± 0.1, *n* = 4	1.28 ± 0.1, *n* = 3
	Roflumilast	1.3 ± 0.1, *n* = 10	**1.2 ± 0.1, *n* = 10** †	1.1 ± 0.1, *n* = 8	1.3 ± 0.1, *n* = 7	1.3 ± 0.1, *n* = 6	1.4 ± 0.1, *n* = 7
**Glucose**	Vehicle	161.5 ± 53.8, *n* = 9	254 ± 142.1, *n* = 9	176.9 ± 74, *n* = 9	193.2 ± 65.1, *n* = 5	114.8 ± 60.2, *n* = 4	144.7 ± 25.4, *n* = 3
	Roflumilast	225.6 ± 75.4, *n* = 10 **	438.3 ± 112.5, *n* = 10 **	**334.8 ± 89.3, *n* = 8** ††	173 ± 24.1, *n* = 7	168.8 ± 18.7, *n* = 6	172.1 ± 21.9, *n* = 7
**Anion GAP**	Vehicle	15.7 ± 4.1, *n* = 8	21.3 ± 6.5, *n* = 8 *	16.3 ± 4.3, *n* = 7	14.3 ± 2.9, *n* = 4	16.3 ± 4.5, *n* = 4	15 ± 3.5, *n* = 3
	Roflumilast	12.8 ± 3.1, *n* = 10	21.1 ± 4.3, *n* = 10 **	16 ± 2.7, *n* = 8	14 ± 1.7, *n* = 7	12.8 ± 3.2, *n* = 6	15 ± 1.9, *n* = 7

Abbreviations: BL, baseline; EOS, end of shock; EOR; end of resuscitation; HR, heart rate. Data is presented as mean ± SD. A Mann–Whitney U test was used for comparisons between the vehicle control and roflumilast treatment groups (†, *p* < 0.05; ††, *p* < 0.01). A paired Wilcoxon signed-rank test was used to compare individual time points to baseline within the vehicle control group (*, *p* < 0.05; **, *p* < 0.01). Statistically significant values are indicated by boldface type.

## Data Availability

The original contributions presented in this study are included in the article. Further inquiries can be directed to the corresponding author.
